# A nomogram for predicting overall survival in patients with muscle-invasive bladder cancer undergoing radical cystectomy: a retrospective cohort study

**DOI:** 10.3389/fonc.2025.1597107

**Published:** 2025-06-19

**Authors:** Haiming Huang, Han Hao, Jialin Du, Lu Pang, Qian Ma, Haixia Li, Lei Jin

**Affiliations:** ^1^ Department of Clinical Laboratory, Peking University First Hospital, Beijing, China; ^2^ Institute of Reproductive and Child Health/National Health Commission Key Laboratory of Reproductive Health, School of Public Health, Peking University, Beijing, China; ^3^ Department of Urology, Peking University First Hospital and Institute of Urology, Beijing, China

**Keywords:** muscle-invasive bladder cancer, radical cystectomy, nomogram, risk factors, overall survival

## Abstract

**Background and aims:**

Radical cystectomy (RC) remains the standard treatment for localized and regionally muscle-invasive bladder cancer (MIBC). However, only half of patients with MIBC survive more than 5 years after RC. We explored the factors associated with overall survival (OS) and constructed a prognostic nomogram for predicting 1-, 3-, and 5-year OS after RC.

**Methods:**

The data were sourced from the Surveillance, Epidemiology, and End Results (SEER) database and Peking University First Hospital (PKUFH). Univariate and multivariate Cox regression analyses were performed using the minimum value of the Akaike information criterion to select independent prognostic factors that significantly contributed to patient survival. A prognostic nomogram was designed to predict 1-, 3-, and 5-year OS.

**Results:**

Among the 16,949 patients with MIBC undergoing surgery, 31.15% survived for more than 5 years. The nomogram we created demonstrated satisfactory discriminative ability to predict the survival of MIBC patients with RC, with area under curve (AUC) of 0.939, 0.880 and 0.852 for 1-, 3- and 5-year OS in the testing set. Moreover, the nomogram still exhibited good performance in an externally independent dataset (1-year: AUC=0.970; 3-year: AUC=0.847; 5-year: AUC=0.790). Furthermore, decision curve analyses showed a modest net benefit for the use of the MIBC nomogram in the current cohort compared to the use of American Joint Committee on Cancer staging alone.

**Conclusions:**

A prognostic nomogram was developed and validated to help clinicians evaluate the prognosis of postoperative MIBC patients. The future integration of additional data will likely improve model performance and accuracy for personalized prognostics.

## 1 Introduction

Bladder cancer is the ninth most commonly diagnosed cancer worldwide, with approximately 614,298 new cases and 220,596 deaths in 2022 (1). Of all cases, 25% are muscle invasive with significant risk of mortality. Non-muscle-invasive bladder cancer has a low lethality rate but can progress to muscle-invasive bladder cancer (MIBC) at a rate of up to 40–80% rate within 5 years (2, 3).

MIBC is characterized by high recurrence, metastasis, and poor prognosis, and is associated with a 5-year survival rate of 60–70%. For MIBC patients with distant metastasis, the 5-year survival rate is only 5% (4). This imposes a heavy economic burden on both patients and society.

Radical cystectomy (RC) remains the standard treatment for patients with MIBC (T2–4) (5, 6). However, the 5-year overall survival (OS) rate in male and female patients is 58–66% and at least 30% of patients experience local recurrence within 5 years after surgery. Most recurrences are diagnosed during the first 2 years (7–10). Thus, it is crucial to identify patients with poor prognosis or high risk of recurrence. These patients may need to be considered for adjuvant treatments or followed more closely for the early monitoring of disease progression and treatment.

At present, tumor stage and regional lymph node status are the primary prognostic variables following RC (11). In general, patients with early-stage and low-grade tumors have a better prognosis, while those with advanced-stage and high-grade tumors have a poorer prognosis. Lymph node metastasis is an important indicator of poor prognosis in bladder cancer, typically suggesting advanced disease progression. The 10-year recurrence-free survival for patients without lymph node involvement is 76% for T1-pT3a, 61% for T3b, and 45% for T4, but when lymph nodes are involved, it drops to 34%, regardless of the stage (12).

Nomograms for cancer-specific survival (CSS) following RC have been developed (13, 14) but their wider use cannot be recommended until more data are available. Shariat et al. (13) reported that the accuracy of a nomogram for the prediction of CSS that included pathologic stage, lymph node status, lymph vascular invasion, neoadjuvant chemotherapy, and adjuvant external beam radiotherapy was significantly superior to the staging based on the American Joint Committee on Cancer (AJCC). However, due to it not being applicable to patients with a non-transitional cell carcinoma histology, as well as the limited sample size and lack of a validation group, that model may lack generalizability in clinical practice. In addition, the effects of racial factors on predictive models in MIBC have not been studied.

The novelty of this work lies in the construction and validation of a nomogram that incorporates various clinical characteristics, ethnicities, and social factors for the prediction of OS, based on the Surveillance, Epidemiology, and End Results (SEER) database. In addition, we conducted external validation in a Chinese population to further confirm its clinical applicability.

## 2 Methods

### 2.1 Data source and study population

**Figure 1:**
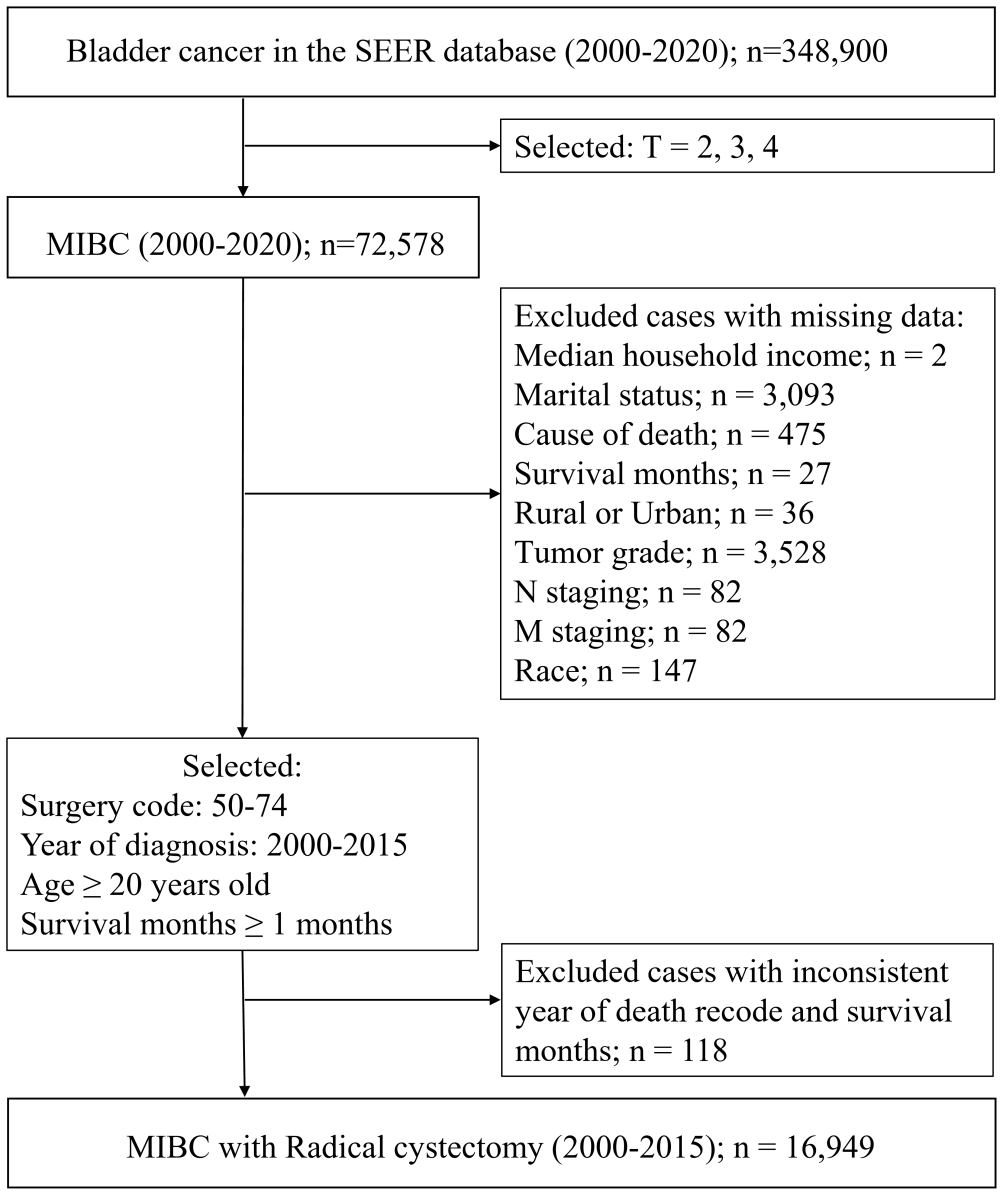
Flow chart of the study.

SEER Database. Data were obtained from 2000–2020 SEER research data covering 17 registries. SEER collects data on cancer diagnoses and survival for approximately 48% of the US population, and it benefits from extensive quality review (15). We selected patients aged 20 years and older who were diagnosed with MIBC and received treatment with RC between January 1, 2000, and December 31, 2015. Patients with missing survival data and those who died within 1 month after initial diagnosis were excluded. Complete data for patient demographics and tumor and treatment variables were retrieved with the use of SEER*Stat version 8.4.3 software. A flow chart for the study procedure is shown in **Figure 1**. This study followed the Strengthening the Reporting of Observational Studies in Epidemiology reporting guidelines.

External validation. Only patients diagnosed with MIBC who underwent RC were included in the study. The same criteria were used to retrospectively collect information from Peking University First Hospital from January 1, 2016, to January 1, 2019, to form an external cohort. Survival status and research variables were determined by telephone or obtained from the most recent medical review by a urologist. Ethical approval was approved by Peking University First Hospital Ethics Committee.

### 2.2 Study variables

Variables were obtained as coded within SEER. Patient demographic variables were age (at diagnosis), sex, race, marital status, household income, and rural or urban residence. Tumor and treatment variables were tumor grade (I, well differentiated; II, moderately differentiated; III, poorly differentiated; and IV, undifferentiated), TNM staging, primary site, and histology. For treatment data, we chose surgery codes 50–74 for RC in the “RX Summ–Surg Prim Site (1998+),” while chemotherapy status was determined as the use of a “chemotherapy recode” in SEER. The endpoint of survival analysis was OS, determined from all causes of death.

### 2.3 Statistical analysis

After cases with insufficient clinicopathologic characteristics were excluded, the remaining eligible patients (n = 16949) were randomly divided into 1:1 training and testing sets. To determine whether there was multicollinearity among the variables, a collinearity diagnosis was performed, and the results showed that the tolerance values for the included variables were less than 1, and the variance impact factor values were all less than 10. A univariate Cox proportional-hazards regression was first performed to screen potential prognostic factors in the training set. Variables with p < 0.1 in univariate Cox regression were considered potential candidate factors for the creation of nomograms. Utilizing bootstrap methods, a series of 1,000 resampling processes undertaken via backward stepwise multivariate Cox regression analyses were conducted, as determined by the Akaike Information Criterion (AIC), to select the best combination of variables. The independent prognostic factors that significantly contributed to patient survival were selected to construct the nomogram, as well as the nomogram for 1-, 3-, and 5-year OS.

The accuracy and discrimination ability of the nomogram across three cohorts (training, testing, and external validation set) were assessed and compared in relation to the calibration curves, concordance index (C-index), and receiver operating characteristic (ROC) curves. To determine clinical practicability, a decision curve analysis (DCA) was performed. The clinicopathological characteristics for patient survival were analyzed by applying the Kaplan–Meier curve and comparing the results using the log-rank test. All analyses were performed using R software (version 4.3.3, R Foundation for Statistical Computing), with the survival package for univariate and multivariable Cox regression analyses, the rms package for the nomogram and calibration curve, the pROC package for the ROC curve, and the ggDCA package for DCA analysis. In each analysis, statistical significance was set at p < 0.05. All analyses were performed using R software (version 4.3.3, R Foundation for Statistical Computing).

## 3 Results

### 3.1 Patient characteristics

From 2000 to 2020, data from a total of 72,578 MIBC patients were drawn from the SEER database. The results indicated an increase in numbers of cases of MIBC per year, while the proportion of patients undergoing RC decreased (**Supplementary Figure S1A**). The survival rate of patients who had undergone RC was nearly twice that of those who do not. The 5-year survival rates for patients who underwent RC and for those who did not were 31% and 15%, respectively (**Supplementary Figure S1B**).

After removing cases that lacked adequate clinicopathological characteristics, the remaining eligible patients (n = 16,949) were randomly divided into training (8,489 patients) and testing (8,489 patients) sets. The baseline characteristics of each cohort are listed in **Table 1**. In addition, 175 patients from the Chinese cohort who met the eligibility criteria were included in this study (**Supplementary Table S1**).

**Table 1:** Baseline characteristics of patients included in the study.

Characteristics	Training set (N=8489)	Testing set (N=8489)	P-value
Sex			0.85
Male	6339 (74.7%)	6327 (74.5%)	
Female	2150 (25.3%)	2162 (25.5%)	
Age			0.29
20–49	422 (5.0%)	425 (5.0%)	
50–59	1399 (16.5%)	1407 (16.6%)	
60–69	2661 (31.3%)	2617 (30.8%)	
70–79	2888 (34.0%)	2826 (33.3%)	
≥80	1119 (13.2%)	1214 (14.3%)	
Race			0.55
White	7577 (89.3%)	7616 (89.7%)	
Black	501 (5.9%)	490 (5.8%)	
Other†	411 (4.8%)	383 (4.5%)	
Marital status			0.51
Married	5565 (65.6%)	5523 (65.1%)	
Other‡	2924 (34.4%)	2966 (34.9%)	
Median household income			0.74
<$45,000	412 (4.9%)	406 (4.8%)	
$45,000–75,000	4766 (56.1%)	4723 (55.6%)	
≥$75,000	3311 (39.0%)	3360 (39.6%)	
Rural Urban			0.47
Metropolitan	7378 (86.9%)	7345 (86.5%)	
Nonmetropolitan	1111 (13.1%)	1144 (13.5%)	
SEER historic stage			0.35
Regional	7886 (92.9%)	7918 (93.3%)	
Distant	603 (7.1%)	571 (6.7%)	
Tumor grade			0.49
II	2986 (35.2%)	3022 (35.6%)	
III	2878 (33.9%)	2805 (33.0%)	
IV	2625 (30.9%)	2662 (31.4%)	
T staging			0.78
II	3424 (40.3%)	3433 (40.4%)	
III	3365 (39.6%)	3326 (39.2%)	
IV	1700 (20.0%)	1730 (20.4%)	
N staging			0.86
N0	6305 (74.3%)	6301 (74.2%)	
N1	1073 (12.6%)	1057 (12.5%)	
N2/N3	1111 (13.1%)	1131 (13.3%)	
M staging			0.08
M0	8076 (95.1%)	8124 (95.7%)	
M1	413 (4.9%)	365 (4.3%)	
Primary site			0.97
Bladder wall regions§	3199 (37.7%)	3186 (37.5%)	
Bladder neck and related structures¶	1937 (22.8%)	1947 (22.9%)	
Structures related to ureters, other$	3353 (39.5%)	3356 (39.5%)	
Histology			0.96
8120–8139: transitional cell papillomas and carcinomas	7606 (89.6%)	7609 (89.6%)	
Other^	883 (10.4%)	880 (10.4%)	0.17
Chemotherapy			
Yes	3363 (39.6%)	3274 (38.6%)	
No/Unknown	5126 (60.4%)	5215 (61.4%)	

†American Indian/Alaska Native, Asian or Pacific Islander.

‡Widowed, Single (never married), Divorced, Separate, Unmarried or Domestic Partner.

§C67.0-Trigone of bladder, C67.1-Dome of bladder, C67.2-Lateral wall of bladder, C67.3-Anterior wall of bladder or C67.4-Posterior wall of bladder.

¶C67.5-Bladder neck or C67.8-Overlapping lesion of bladder.

$C67.6-Ureteric orifice, C67.7-Urachus or C67.9-Bladder, NOS.

^8000-8009: unspecified neoplasms, 8010-8049: epithelial neoplasms, NOS, 8050-8089: squamous cell neoplasms, 8090-8119: basal cell neoplasms, 8140-8389: adenomas and adenocarcinomas, 8440-8499: cystic, mucinous and serous neoplasms, 8500-8549: ductal and lobular neoplasms or 8560-8579: complex epithelial neoplasms.

Among the MIBC patients in the training set, the majority were male, accounting for about 74.7%. The age distribution ranged from 20 to over 80 years, with 34% of the patients between 70 and 79 years old. White people formed the vast majority of patients (89.3%), followed by Black people (5.9%) and other races, including American Indian, Alaska Native, and Asian or Pacific Islander (4.8%). In terms of marital status, most were married (65.6%). Tumor characteristics, including tumor grade, TNM staging, primary site, and histology showed an even distribution between the two groups without significant deviation. Regarding chemotherapy, about 39.6% of the patients chose to undergo treatment, while around 60.4% did not receive chemotherapy or had no record of receiving it.

### 3.2 Risk factors associated with OS

The detailed results of the univariate Cox regression analysis of the training set are presented in **Table 2**. Sex, age, race, marital status, tumor grade, TNM staging, primary site, histology, and chemotherapy were all significantly related to OS. In a subsequent multivariate Cox regression, all of these significant factors were first incorporated into a Cox regression model. To select independent prognostic factors that significantly contributed to patient survival and that could be included in the nomogram, we conducted 1,000 bootstrap resampling processes of the AIC for variable selection. Ultimately, the key factors for predicting OS were determined, including sex, age, race, marital status, tumor grade, TNM staging, histology, and chemotherapy (**Table 2**).

**Table 2:** Results of univariate and multivariate Cox analyses for OS of MIBC patients with RC in the training set.

Characteristics	Univariate		Multivariate (all)		Multivariate (1000AIC-based model)	
	HR (95% CI)	P-value	HR (95% CI)	P-value	HR (95% CI)	P-value
Sex						
Male	Reference		Reference		Reference	
Female	1.062 (1.003–1.124)	0.038	0.9276 (0.8736–0.9849)	1.40E-02	0.8735 (0.8218–0.9283)	<0.001
Age						
20–49	Reference		Reference		Reference	
50–59	0.985 (0.856–1.133)	0.831	1.0485 (0.9112–1.2065)	0.508	0.9702 (0.8429–1.1167)	0.673
60–69	1.315 (1.154–1.499)	<0.001	1.425 (1.2494–1.6253)	<0.001	1.3837 (1.2127–1.5788)	<0.001
70–79	1.718 (1.509–1.955)	<0.001	1.8283 (1.6044–2.0833)	<0.001	1.7356 (1.5226–1.9784)	<0.001
≥80	2.335 (2.035–2.681)	<0.001	2.3901 (2.0777–2.7495)	<0.001	2.3415 (2.0336–2.6959)	<0.001
Race						
White	Reference		Reference		Reference	
Black	1.168 (1.053–1.295)	0.003	1.1121 (1.001–1.2355)	4.79E-02	1.2032 (1.0825–1.3372)	<0.001
Other†	0.898 (0.796–1.014)	0.082	0.8779 (0.7779–0.9908)	0.0349	0.9872 (0.8752–1.1135)	0.834
Marital status						
Married	Reference		Reference		Reference	
Other‡	1.258 (1.195–1.325)	<0.001	1.2053 (1.1417–1.2725)	<0.001	1.2017 (1.1372–1.2698)	<0.001
Median household income						
<$45,000	Reference					
$45,000–75,000	0.944 (0.839–1.060)	0.329				
≥$75,000	0.902 (0.801–1.016)	0.09				
**Rural Urban**						
Metropolitan	Reference					
Nonmetropolitan	1.053 (0.979–1.132)	0.163				
Tumor grade						
II	Reference		Reference		Reference	
III	1.862 (1.749–1.983)	<0.001	1.0055 (0.8759–1.1543)	0.938	0.9811 (0.8541–1.1269)	0.787
IV	2.742 (2.573–2.922)	<0.001	1.2024 (1.0183–1.4198)	0.03	1.2449 (1.0541–1.4702)	0.0098
T staging						
II	Reference		Reference		Reference	
III	1.926 (1.818–2.041)	<0.001	1.6891 (1.4891–1.916)	<0.001	1.6567 (1.4603–1.8795)	<0.001
IV	2.722 (2.545–2.912)	<0.001	2.1791 (1.9167–2.4774)	<0.001	2.3043 (2.0256–2.6214)	<0.001
N staging						
N0	Reference		Reference		Reference	
N1	1.667 (1.550–1.792)	<0.001	1.3066 (1.1511–1.483)	<0.001	1.247 (1.0996–1.4142)	<0.001
N2/N3	2.209 (2.060–2.370)	<0.001	1.6919 (1.4953–1.9144)	<0.001	1.4866 (1.3148–1.6809)	<0.001
M staging						
M0	Reference		Reference		Reference	
M1	2.392 (2.152–2.660)	<0.001	1.602 (1.4291–1.7959)	<0.001	1.6097 (1.4394–1.8001)	<0.001
Primary site						
Bladder wall regions§	Reference		Reference			
Bladder neck and related structures*	1.135 (1.062–1.212)	<0.001	1.0421 (0.9748–1.1139)	0.226		
Structures related to ureters, others$	1.136 (1.073–1.202)	<0.001	1.0726 (1.013–1.1357)	0.0162		
Histology						
8120–8139: transitional cell papillomas and carcinomas	Reference		Reference		Reference	
Other^	1.248 (1.154–1.350)	<0.001	1.2049 (1.1128–1.3046)	<0.001	1.2503 (1.1558–1.3525)	<0.001
Chemotherapy						
Yes	Reference		Reference		Reference	
No/Unknown	1.155 (1.096–1.216)	<0.001	1.2922 (1.222–1.3665)	<0.001	1.2978 (1.2273–1.3723)	<0.001

†American Indian/Alaska Native, Asian or Pacific Islander.

‡Widowed, single (never married), divorced, separated, unmarried, or with domestic partner.

§C67.0, trigone of bladder; C67.1, dome of bladder; C67.2, lateral wall of bladder; C67.3, anterior wall of bladder; or C67.4, posterior wall of bladder.

¶C67.5, bladder neck, or C67.8, overlapping lesion of bladder.

$C67.6, ureteric orifice; C67.7, urachus; or C67.9, bladder, NOS.

^8000–8009: unspecified neoplasms, 8010–8049: epithelial neoplasms, NOS, 8050–8089: squamous cell neoplasms, 8090–8119: basal cell neoplasms, 8140–8389: adenomas and adenocarcinomas, 8440–8499: cystic, mucinous, and serous neoplasms, 8500–8549: ductal and lobular neoplasms, or 8560–8579: complex epithelial neoplasms.

The Kaplan-Meier survival curves for OS are displayed in **Supplementary Figure S2**. Female, age > 60 years, unmarried status, high tumor grade and TNM staging, diagnosis of non-transitional cell carcinoma, and absence of chemotherapy were associated with poor survival prognosis.

Interestingly, in a Cox regression analysis, we found an interaction between age and sex. Between the ages of 20 and 60 years, males demonstrated a higher survival probability than females, suggesting a protective effect of sex on survival in this period. However, this pattern reversed over time, and after 80 years old, the probability of survival for females exceeded that of males. Nonetheless, overall, being of the male sex remains a protective factor. This reveals the dynamic changes in the effect of sex on survival time with age (**Supplementary Figure S3**).

### 3.3 Nomogram construction and validation

**Figure 2:**
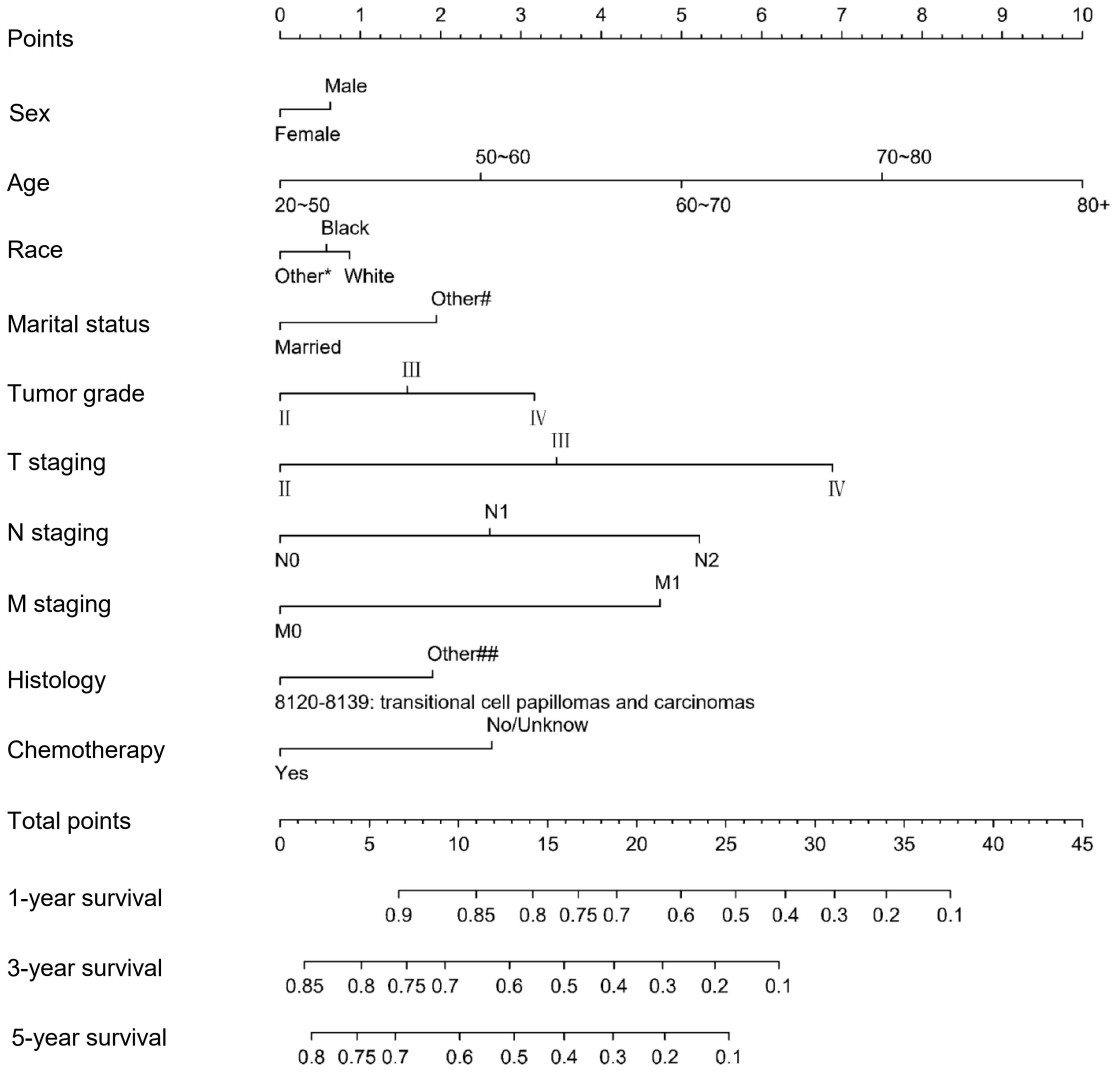
Nomogram for predicting 1-, 3-, and 5-year overall survival for MIBC patients with RC in the training set.

**Figure 3:**
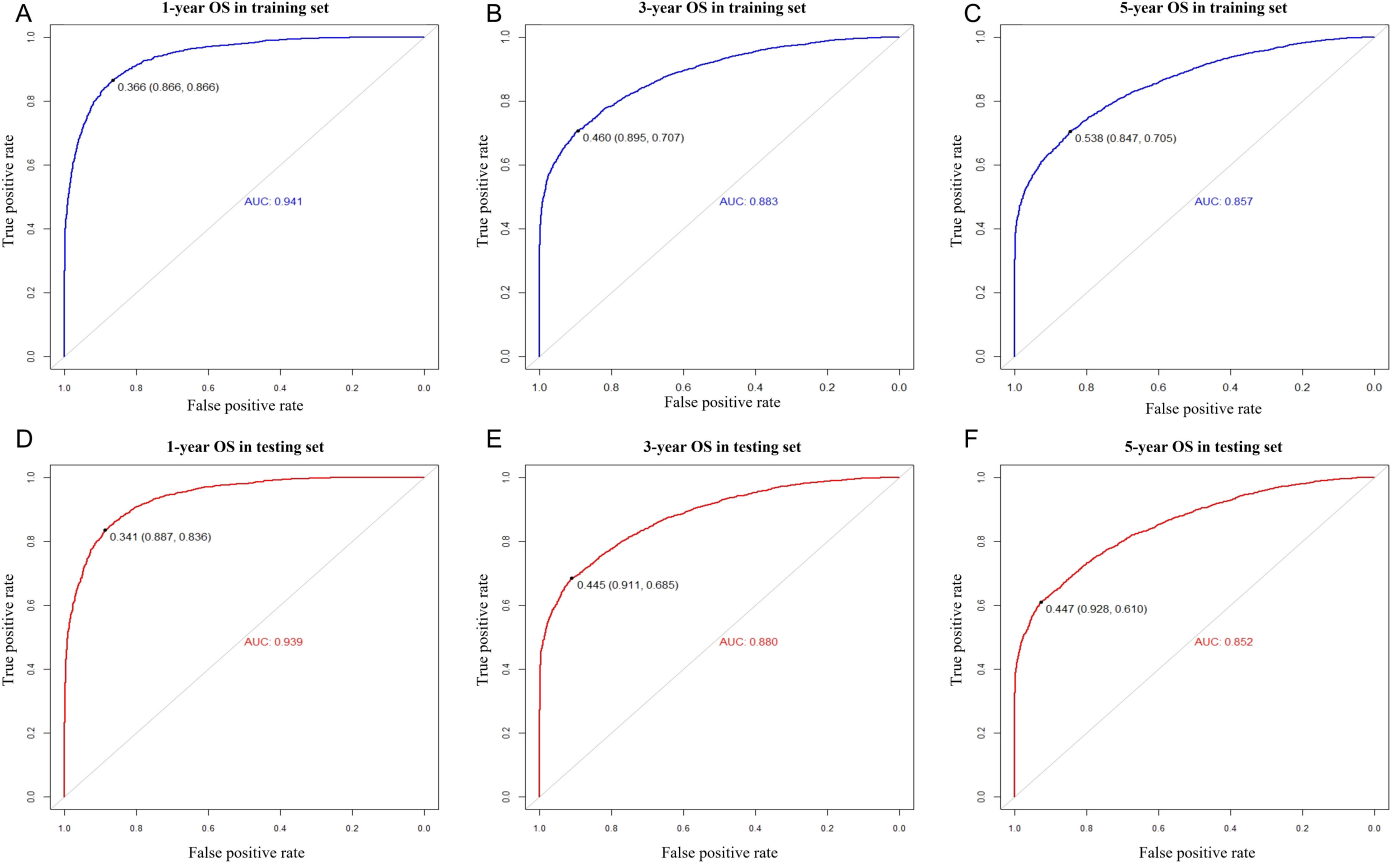
ROC curves for predicting 1-, 3-, and 5-year overall survival for MIBC patients with RC in the training set **(A–C)** and the testing set **(D–F)**.

We included the above factors as potential predictors for the 1-, 3-, and 5-year OS in the training set to present a nomogram graph as shown in **Figure 2**. The 1-, 3-, and 5-year calibration curves for the training and testing sets did not show large fluctuations, indicating that the predicted results were basically consistent with the actual results in the two cohorts, and the model had good accuracy (**Supplementary Figure S4**). The C-index of the nomogram used to estimate OS in the training set was 0.671 (95% confidence interval [CI] 0.663–0.679). The AUC values of the 1-, 3-, and 5-year OS were 0.941, 0.883, and 0.857 in the training set. For the 1-, 3-, and 5-year OS of the testing set, the nomogram demonstrated favorable discrimination, with AUC values of 0.939, 0.880, and 0.852, respectively. The C-index of the nomogram used to predict OS was 0.672 (95% CI 0.664–0.680), indicating that the prediction model had good discrimination (**Figure 3**).

After resolving the accuracy of the model, the testing set was used to perform DCA to make the nomogram clinically practical. The nomogram showed had high clinical potential for the prediction of OS, with a wide and practical threshold probability range through 1-, 3-, and 5-year OS. Moreover, it outperformed the AJCC TNM staging system because more clinical net benefits were obtained within a wider threshold probability range using it (**Supplementary Figure S5**), meaning that our nomogram can aid clinical decision-making and predict outcomes for patients.

**Figure 4:**
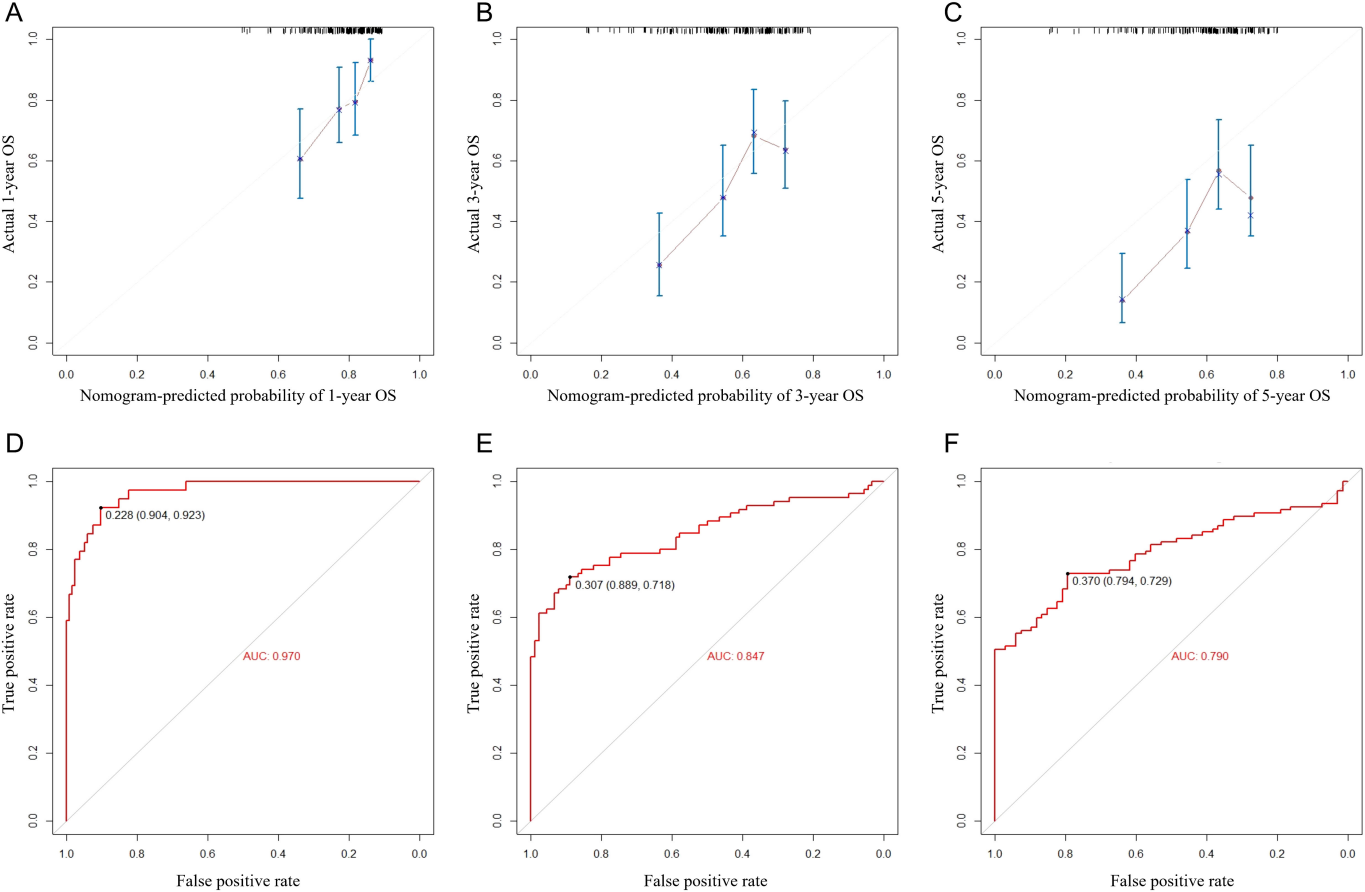
Calibration curves **(A–C)** and ROC curves **(D–F)** for predicting 1-, 3-, and 5-year overall survival for MIBC patients with RC in the external validation set.

Finally, we tested the nomogram in a Chinese cohort (Peking University First Hospital, 2016–2019) and found that it maintained good discrimination and consistency in the Asian population. Specifically, the C-index reached 0.631 (95% CI 0.533–0.729), and the AUC values for 1-, 3-, and 5-year OS were 0.970, 0.847, and 0.790. These indicators suggest that the nomogram has relatively good predictive power, which makes it suitable for clinical practice in Asian populations (**Figure 4**).

## 4 Discussion

RC with pelvic lymphadenectomy is the most effective and most widely used definitive local treatment for invasive bladder cancer. In this study, we selected 72,578 MIBC patients from the SEER database; the results showed an increasing incidence of MIBC per year, with the highest incidence in patients aged 70–79 years. As found in previous studies, 50% of MIBC patients who underwent RC had improved survival rates (16, 17). However, postoperative morbidity remains high, partly because of postoperative complications (such as ureteric reflux and the deterioration of renal function, chronic/recurrent pyelonephritis, and metabolic disorders) or recurrent bladder cancer (18, 19). The reported OS rates after RC in elderly patients (≥70 year) range between 36% and 66% at 3 years and between 8% and 54% at 5 years (18). Therefore, it is imperative to investigate the prognostic factors that affect the survival of patients undergoing surgery to provide an estimate of the risk of disease progression in MIBC patients undergoing RC.

In recent years, several researchers have contributed to the development of postoperative predictive models for bladder cancer. A study based on data from more than 9,000 bladder cancer patients from 12 centers worldwide constructed a database that contained patient characteristics and pathological details. They developed an international bladder cancer nomogram that predicted recurrence risk for bladder cancer after RC. Although the accuracy of that study nomogram is significantly better than commonly used standards, it does not contain treatment information. It simply provides a way to assess the risk of disease recurrence in an individual patient after RC assuming no adjuvant therapy is received (20). Based on demographic, pathological, and treatment information on 640 patients, Maria et al. (21) developed a unique real-world tool for the prediction of bladder cancer death in patients with MIBC who had received NAC before RC. However, it remains unclear whether other treatment regimens and demographic characteristics, such as marital status, income, and race, can be used to predict postoperative survival.

In this study, 16,496 MIBC patients who received RC treatment were selected from the SEER database, who were randomized to the training and testing sets at a 1:1 ratio. Based on multivariate Cox regression analysis, sex, age, race, marital status, tumor grade, TNM staging, histology, and chemotherapy were identified as independent prognostic predictors of OS. On the basis of these factors, we developed a nomogram to predict the OS of MIBC patients undergoing RC for 1, 3, and 5 years and further evaluated its performance. The findings suggest that older age, being male, and of the Black people race are epidemiological features associated with poor MIBC prognosis. Bladder cancer was found to more commonly affect older individuals, with >78% of cases occurring in ages > 60 years. Several studies with large sample sizes have demonstrated a significant relationship between age at RC and perioperative complication rate (18, 22, 23). Other factors, such as a long history of smoking, underlying health problems, and late diagnosis and treatment limitations, may also contribute to lower OS in older patients. Males are more commonly affected by bladder cancer, with a male-to-female ratio of approximately 3:1 in the training and testing sets. Several hypotheses have been proposed for this, including differences in tobacco use and exposure to specific compounds in the workplace, hormonal factors, and the influence of sex chromosomes (24–26). Interestingly, in this study, we noted an interaction between age and sex, indicating that as age increases, the difference in 5-year OS rates between males and females become less significant. Further exploration and validation are required to corroborate these findings. In addition, we found that unmarried patients, including widowed patients, are at significantly greater risk for poor prognosis and mortality than married patients. This may be due to the fact that married patients are less likely to develop metastatic disease and are more likely to receive definitive therapy (27). In line with previous studies, we found that OS was better in people with low-grade, early-stage tumors than in those with high-grade, advanced-stage tumors (11, 20, 28). About 90% of cases of bladder cancer are transitional cell papillomas and carcinomas (TCCs) (3), and 10% are squamous cell neoplasms and other histology subtypes. In this study, we found that having TCC was an advantage for patients undergoing RC. Several studies have shown that SCC accounts for <3% of all newly diagnosed cases of bladder cancer in the United States, and is more aggressive and lethal than urothelial cancer (29, 30). In addition, our study confirms that chemotherapy for MIBC is associated with improved OS (21). Furthermore, the results of C-index and AUC analyses demonstrated strong concordance with our nomogram. Indeed, the DCA results validate its clinical utility. Therefore, the variables used to construct the prognostic model in this study are statistically reliable.

Our study also had several limitations. First, the external validation cohort consisted of single-center data, with certain characteristics, such as race and marital status, being relatively uniform. Therefore, it is essential to perform further validation through prospective multicenter clinical trials. Second, the accuracy of the model should be further improved; more risk factors related to OS could be included in the future, such as preoperative laboratory results and tumor markers other than PD1/PDL1, which may also be significant predictors and prognostic indicators for MIBC disease progression and treatment resistance. Finally, the SEER database also lacks information on specific treatment plans, such as adjuvant therapy and neoadjuvant therapy. Further analysis is needed to evaluate their impact on patient prognosis.

## 5 Conclusions

In summary, a nomogram was constructed and validated from diverse groups of MIBC patients. Compared to the conventional staging system, our nomogram offers increased accuracy, good clinical utility, and more precise prognosis for 1-, 3-, and 5-year OS. It provides an accurate risk assessment for postoperative MIBC patients.

## Data Availability

The original contributions presented in the study are included in the article/**Supplementary Material**. Further inquiries can be directed to the corresponding authors.

## Supplementary material

Supplementary Figure 1 Incidence of MIBC in the SEER database **(A)** and survival rates between MIBC with and without RC **(B)**.

Supplementary Figure 2 The clinicopathologic characteristics on patients’ overall survival of MIBC patients with RC in training set and the results suggested that sex**(A)**, age**(B)**, race**(C)**, marital status**(D)**, tumor grade**(E)**, T staging**(F)**, N staging**(G)**, M staging**(H)**, primary site**(I)**, histology**(G)** and chemotherapy**(K)** was significance impact patients’ prognostic.

Supplementary Figure 3 Differences in 5-year survival between males and females across different age groups aged 20–50 years **(A)**, 50–60 years **(B)**, 60–70 years **(C)**, 70–80 years **(D)**, 80+ years **(E)**, and all ages **(F)**.

Supplementary Figure 4 Calibration curves for predicting 1-, 3-, and 5-year overall survival for MIBC patients with RC in the training set **(A–C)** and the testing set **(D–F)**.

Supplementary Figure 5 Decision curve analysis between the nomogram and the AJCC staging system for 1-, 3-, and 5-year overall survival of MIBC patients with RC in the testing set.
