# Supplementary material for: A nomogram for predicting overall survival in patients with muscle-invasive bladder cancer undergoing radical cystectomy: a retrospective cohort study
Source: Front Oncol. 2025 Jun 19;15:1597107. doi: 10.3389/fonc.2025.1597107 (PMC12221893; doi:10.3389/fonc.2025.1597107)
Supplement: Supplementary Figure 1 — Incidence of MIBC in the SEER database (A) and survival rates between MIBC with and without RC (B). [file SupplementaryFile1.zip › Supplementary Figures/Supplementary Figures.docx]

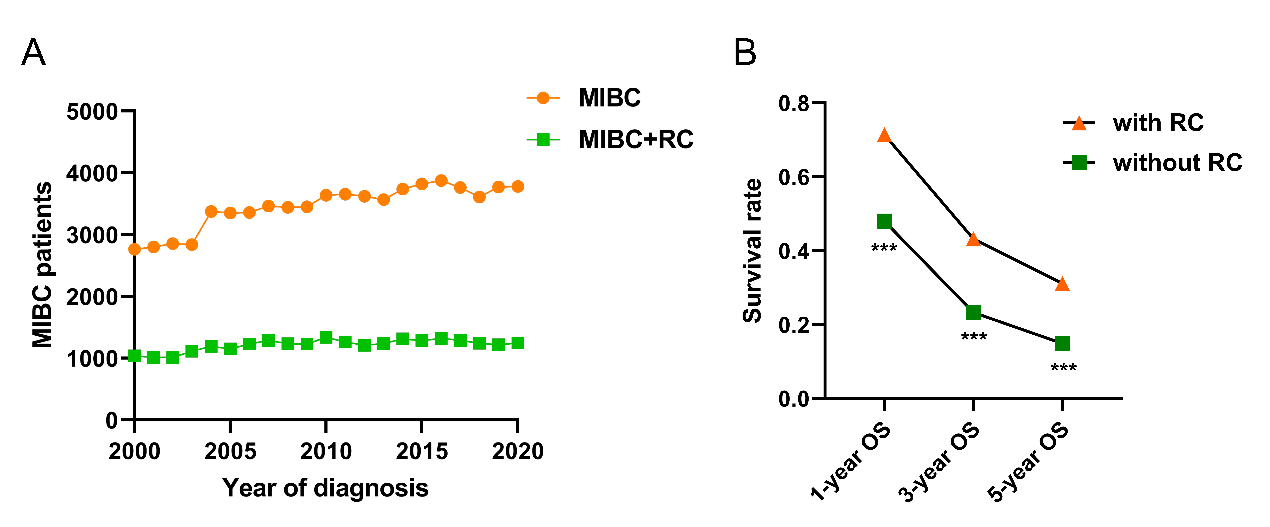


**Supplementary Figure S1.** Incidence of MIBC in the SEER database (A) and survival rates between MIBC with and without RC (B).

**
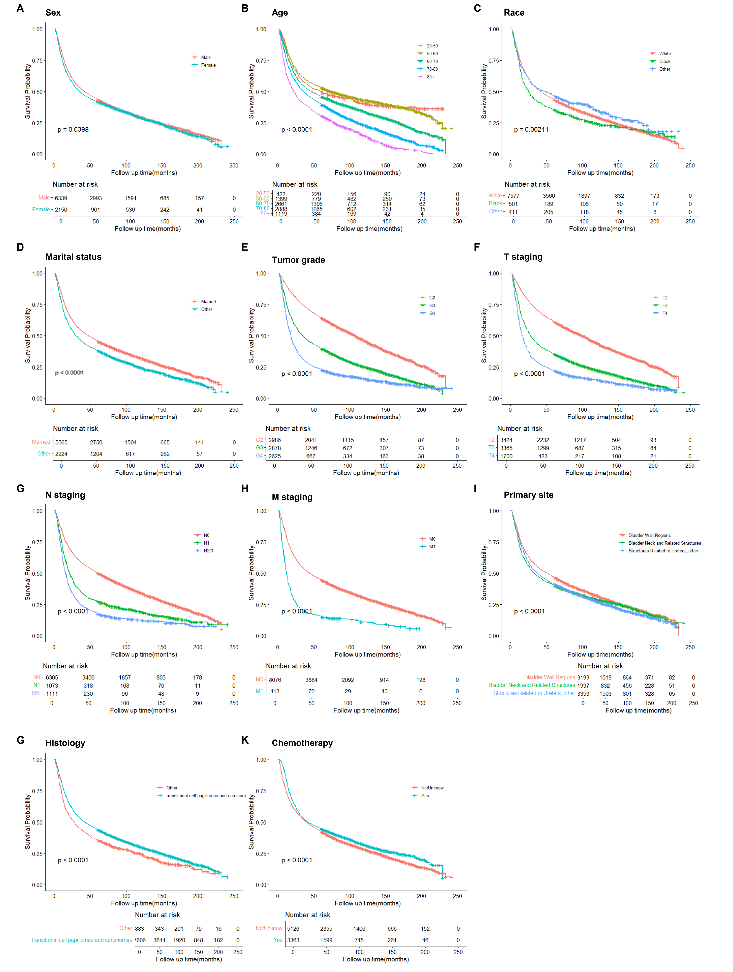
**

**Supplementary Figure S2.** The clinicopathologic characteristics on patients’ overall survival of MIBC patients with RC in training set and the results suggested that sex(A), age(B), race(C), marital status(D), tumor grade(E), T staging(F), N staging(G), M staging(H), primary site(I), histology(G) and chemotherapy(K) was significance impact patients’ prognostic.

**
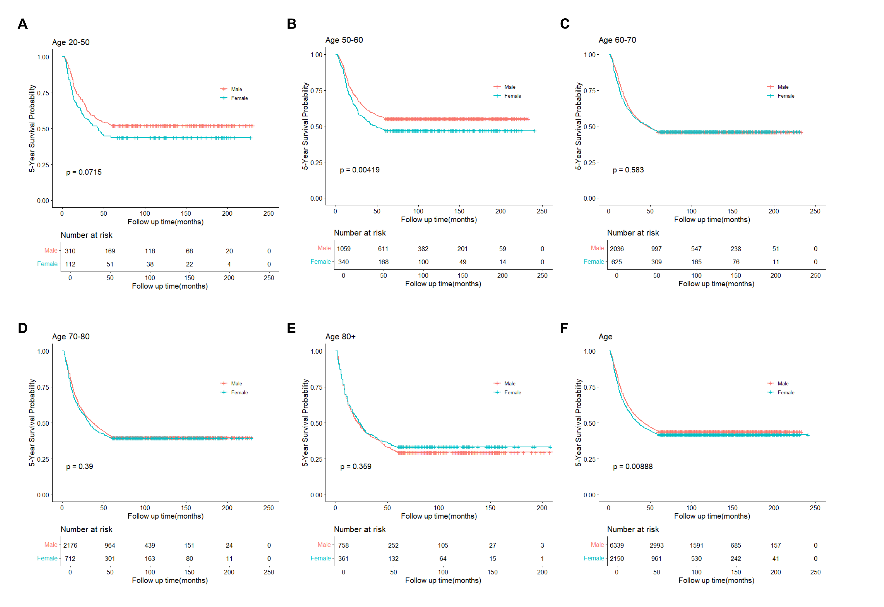
**

**Supplementary Figure S3.** Differences in 5-year survival between males and females across different age groups aged 20–50 years (A), 50–60 years (B), 60–70 years (C), 70–80 years (D), 80+ years (E), and all ages (F).


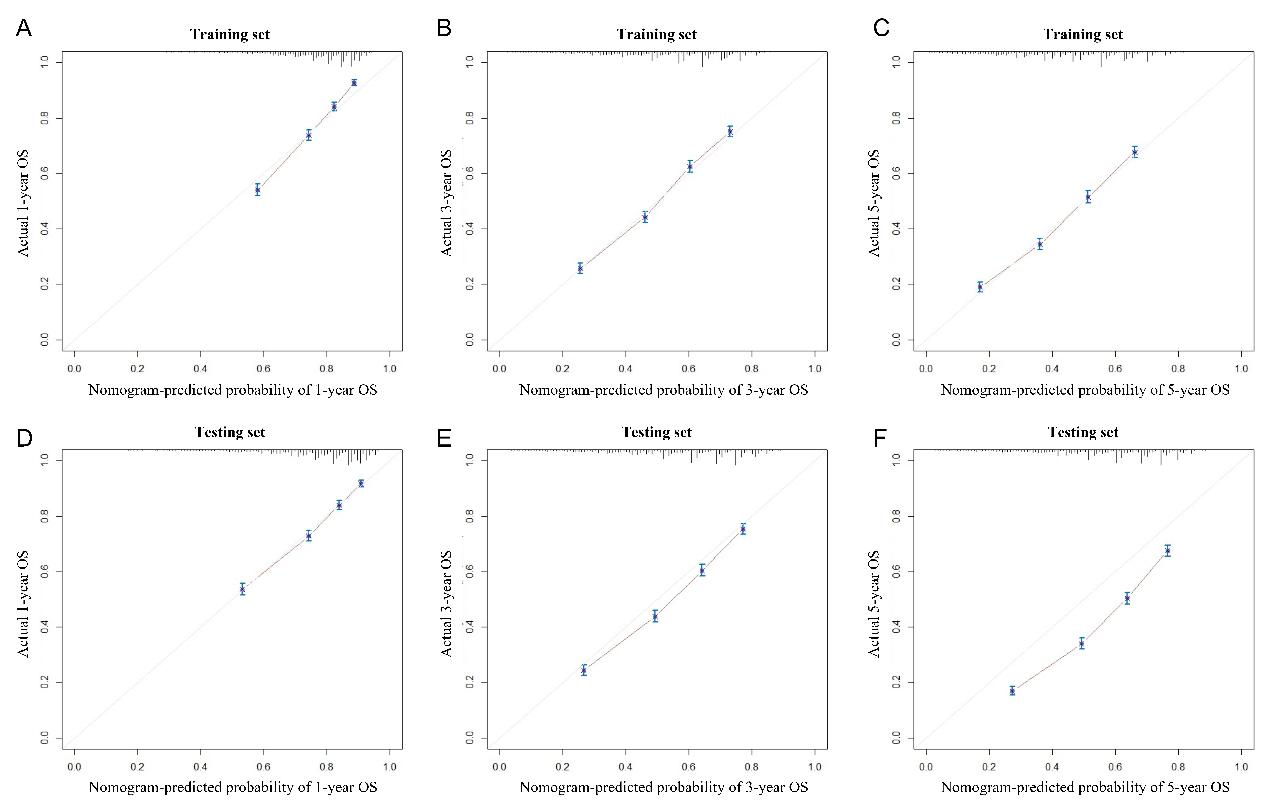


**Supplementary Figure S4.** Calibration curves for predicting 1-, 3-, and 5-year overall survival for MIBC patients with RC in the training set (A–C) and the testing set (D–F).


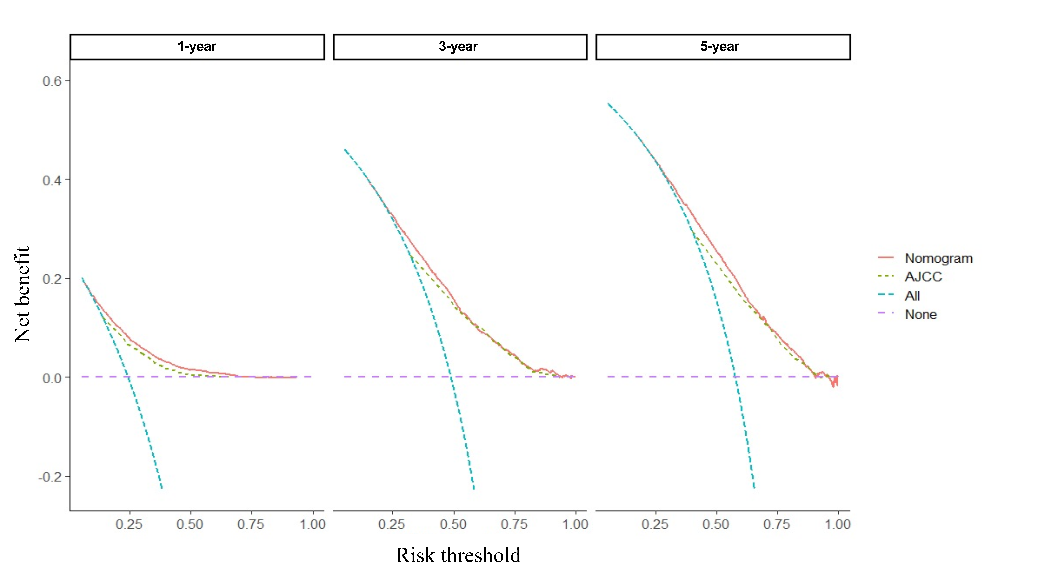


**Supplementary Figure S5**. Decision curve analysis between the nomogram and the AJCC staging system for 1-, 3-, and 5-year overall survival of MIBC patients with RC in the testing set.
