# Supplementary figures and images for: A nomogram for predicting overall survival in patients with muscle-invasive bladder cancer undergoing radical cystectomy: a retrospective cohort study
Source: Front Oncol. 2025 Jun 19;15:1597107. doi: 10.3389/fonc.2025.1597107 (PMC12221893; doi:10.3389/fonc.2025.1597107)

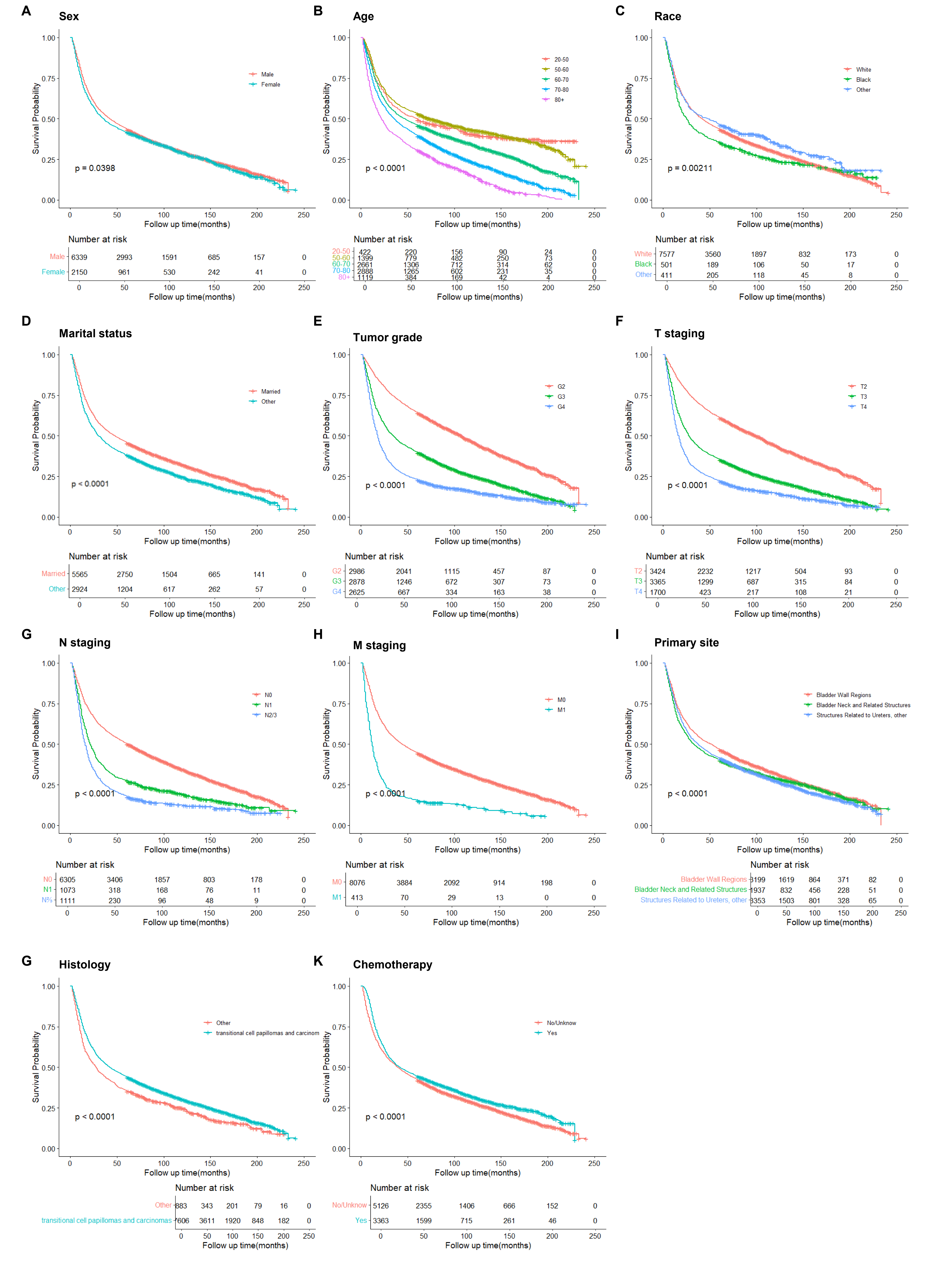

Supplement: Supplementary Figure 1 — Incidence of MIBC in the SEER database (A) and survival rates between MIBC with and without RC (B). [file SupplementaryFile1.zip › Supplementary Figures/Supplementary Figure S2.TIF]

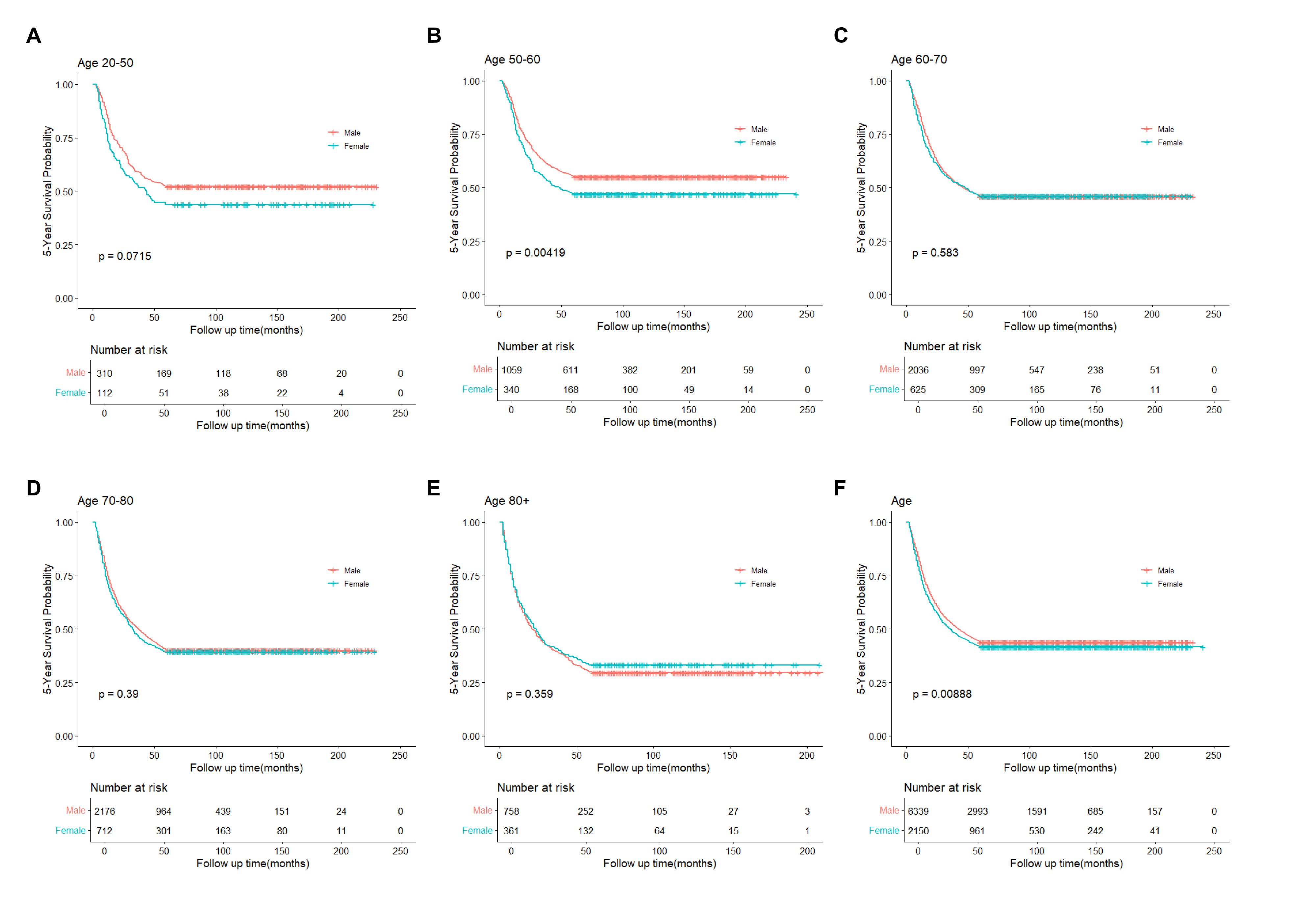

Supplement: Supplementary Figure 1 — Incidence of MIBC in the SEER database (A) and survival rates between MIBC with and without RC (B). [file SupplementaryFile1.zip › Supplementary Figures/Supplementary Figure S3.TIF]

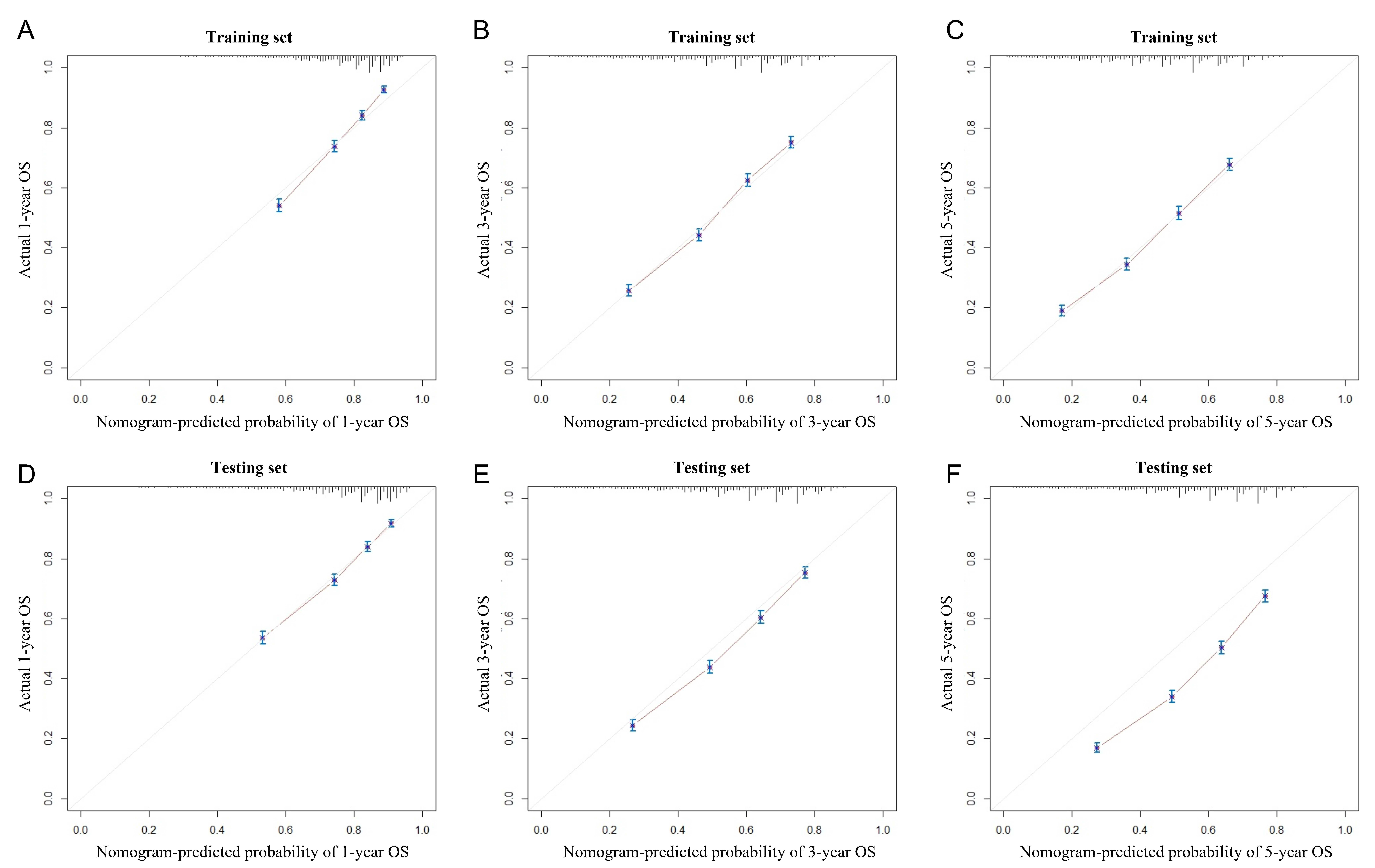

Supplement: Supplementary Figure 1 — Incidence of MIBC in the SEER database (A) and survival rates between MIBC with and without RC (B). [file SupplementaryFile1.zip › Supplementary Figures/Supplementary Figure S4.TIF]
