# Supplementary material for: A nomogram for predicting overall survival in patients with muscle-invasive bladder cancer undergoing radical cystectomy: a retrospective cohort study
Source: Front Oncol. 2025 Jun 19;15:1597107. doi: 10.3389/fonc.2025.1597107 (PMC12221893; doi:10.3389/fonc.2025.1597107)
Supplement: Supplementary file 2 [file Table1.docx]

| **Supplementary Table S1. Baseline characteristics of patients included in the study** | |
| --- | --- |
| **Characteristics** | **External validation set (N = 175)** |
| **Sex** |  |
| Male | 139 (79.4%) |
| Female | 36 (20.6%) |
| **Age** |  |
| 20-49 | 16.0 (9.1%) |
| 50-59 | 43.0 (24.6%) |
| 60-69 | 50.0 (28.6%) |
| 70-79 | 53.0 (30.3%) |
| ≥80 | 13.0 (7.4%) |
| **Race** |  |
| Aisan | 175 (100.0%) |
| Other | 0 |
| **Marital status** |  |
| Married | 173 (98.9%) |
| Other ^†^ | 2 (1.1%) |
| **Tumor grade** |  |
| Ⅱ | 35 (20.0%) |
| Ⅲ | 140 (80.0%) |
| Ⅳ | 0 |
| **T staging** |  |
| Ⅱ | 69.0 (39.4%) |
| Ⅲ | 65.0 (37.1%) |
| Ⅳ | 41.0 (23.4%) |
| **N staging** |  |
| N0 | 137.0 (78.3%) |
| N1 | 11.0 (6.3%) |
| N2/N3 | 27.0 (15.4%) |
| **M staging** |  |
| M0 | 126.0 (72.0%) |
| M1 | 49.0 (28.0%) |
| **Histology** |  |
| 8120-8139: transitional cell papillomas and carcinomas | 172.0 (98.3%) |
| Other ^‡^ | 3.0 (1.7%) |
| **Chemotherapy** |  |
| Yes | 53.0 (30.3%) |
| No/Unknow | 122.0 (69.7%) |
| ^†^Single (never married), Divorced. | |
| ^‡^8000-8009: unspecified neoplasms, 8010-8049: epithelial neoplasms, NOS, 8050-8089: squamous cell neoplasms, 8090-8119: basal cell neoplasms, 8140-8389: adenomas and adenocarcinomas, 8440-8499: cystic, mucinous and serous neoplasms, 8500-8549: ductal and lobular neoplasms or 8560-8579: complex epithelial neoplasms. | |
